# Innovative Microencapsulation of Polymyxin B for Enhanced Antimicrobial Efficacy via Coated Spray Drying

**DOI:** 10.1021/acs.molpharmaceut.4c00594

**Published:** 2024-10-08

**Authors:** Amal Yousfan, Arwa Omar Al Khatib, Afrah M. H. Salman, Mahmoud H. Abu Elella, Glyn Barrett, Nicholas Michael, Mohammed Gulrez Zariwala, Hisham Al-Obaidi

**Affiliations:** † School of Pharmacy, University of Reading, Reading RG6 6AD, U.K.; ‡ Faculty of Pharmacy, Al Ahliyya Amman University, Amman 19111, Jordan; § School of Biological Sciences, University of Reading, Reading RG6 6AD, U.K.; ∥ College of Pharmacy, Pharmacology and Toxicology Department, Mustansiriyha University, Baghdad 14132, Iraq; ⊥ Chemical Analysis Facility, University of Reading, Reading RG6 6AD, U.K.; # Centre for Nutraceuticals, School of Life Sciences, University of Westminster, 115 New, Cavendish Street, London W1W 6UW, U.K.

**Keywords:** polymyxin B, microencapsulation, three-fluid nozzle spray drying, core/shell structure, Pseudomonas aeruginosa, antibiofilm assay

## Abstract

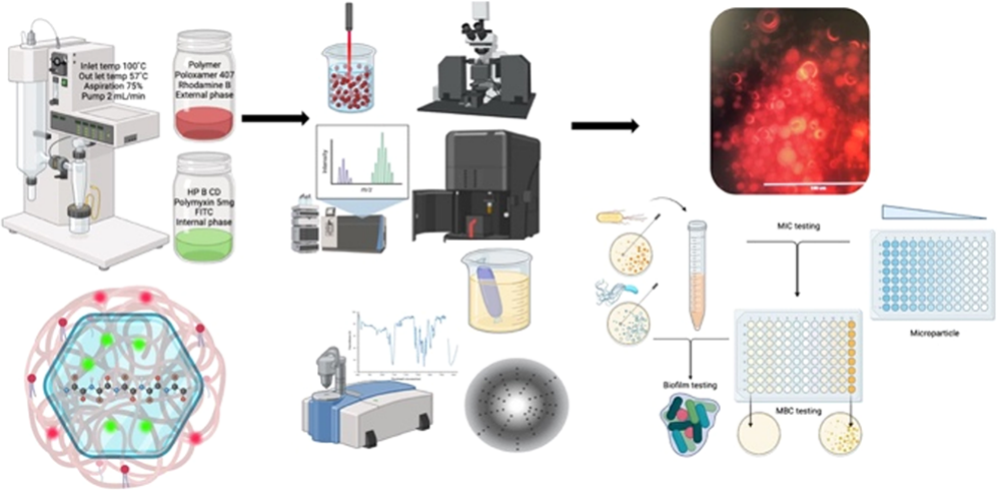

This study aims to develop an innovative microencapsulation method for coated Polymyxin B, utilizing various polysaccharides such as hydroxypropyl β-cyclodextrin, alginate, and chitosan, implemented through a three-fluid nozzle (3FN) spray drying process. High-performance liquid chromatography (HPLC) analysis revealed that formulations with a high ratio of sugar cage, hydroxypropyl β-cyclodextrin (HPβCD), and sodium alginate (coded as ALG_H_CD_H_P_L_^PM^) resulted in a notable 16-fold increase in Polymyxin B recovery compared to chitosan microparticles. Morphological assessments using fluorescence labeling confirmed successful microparticle formation with core/shell structures. Alginate-based formulations exhibited distinct layers, while chitosan formulations showed uniform fluorescence throughout the microparticles. Focused beam reflectance and histograms from fluorescence microscopic measurements provided insights into physical size analysis, indicating consistent sizes of 6.8 ± 1.2 μm. Fourier-transform infrared (FTIR) spectra unveiled hydrogen bonding between Polymyxin B and other components within the microparticle structures. The drug release study showed sodium alginate’s sustained release capability, reaching 26 ± 3% compared to 94 ± 3% from the free solution at the 24 h time point. Furthermore, the antimicrobial properties of the prepared microparticles against two Gram-negative bacteria, *Escherichia coli* and *Pseudomonas aeruginosa*, were investigated. The influence of various key excipients on the minimum inhibitory concentration (MIC) and minimum bactericidal concentration (MBC) values was evaluated. Results demonstrated effective bactericidal effects of ALG_H_CD_H_P_L_^PM^ against both *E. coli* and *P. aeruginosa*. Additionally, the antibiofilm assay highlighted the potential efficacy of ALG_H_CD_H_P_L_^PM^ against the biofilm viability of *E. coli* and *P. aeruginosa*, with concentrations ranging from 3.9 to 500 μg/m. This signifies a significant advancement in antimicrobial drug delivery systems, promising improved precision and efficacy in combating bacterial infections.

## 1 Introduction

A current key driver of the pharmaceutical industry is the research and development of drug delivery systems. Efforts are specifically centered on antibiotics, including Polymyxin B and Polymyxin E (colistin), which are renowned for their effectiveness against various Gram-negative bacterial pathogens.^1^ Despite its critical role as a last resort antibiotic for tackling multidrug-resistant bacteria, Polymyxin B faces significant challenges in clinical application and the uncertainty surrounding optimal dosing strategies.^1^

Polymyxin B, a cationic polypeptide antibiotic derived from *Paenibacillus polymyxa* fermentation, can be administered intravascularly, intrathecally, or topically as Polymyxin B sulfate. Oral administration is generally avoided due to poor bioavailability.^1^ The core challenges faced by Polymyxin originate from stability issues, including hydrolysis and degradation under diverse conditions, posing a substantial threat to its therapeutic effectiveness.^2^ Additionally, there are concerns regarding potential systemic nephrotoxicity and neurotoxicity upon their systematic administration. To enhance the performance of Polymyxin B, various drug delivery systems have been developed. Mesoporous silica nanoparticles loaded with tannic acid and Polymyxin B offer pH-sensitive and sustained release. Liposomes and niosomes improve bioavailability and reduce toxicity, while conjugates such as polymyxin–cinnamaldehyde and dextrin–colistin modify drug properties to combat microbial resistance. Hydrogels, microgels, electrospun nanofibers, and elastomer nanocomposite membranes extend the antimicrobial activity and support wound healing. Additionally, microneedles enable minimally invasive transdermal delivery, ensuring efficient antibiotic release and efficacy. Despite promising results, scaling up colistin-loaded nanostructured lipid carriers, which show potential in reducing colistin toxicity, remains a challenge.^3,4^

In response to these hurdles, our primary focus addresses the encapsulation of Polymyxin B within polymeric saccharide microparticles to increase the effectivity for lung delivery. This strategic shift aims to optimize the efficacy of Polymyxin B while minimizing potential adverse effects in clinical use.^5^

Spray drying is a widely used technique in pharmaceutical formulation that turns liquid solutions into solid powders. This process breaks down the liquid into fine droplets and quickly dries them into solid particles. The benefits of spray-dried peptide products are significant, mainly due to improved stability achieved by removing water.^6^ This reduces mobility and slows down degradation. Peptides stabilized within sugar glass, formed using spray drying with sugars like 2-hydroxypropyl β-cyclodextrin (HPβCD), are known for their inertness and rapid solubility.^7^

Two theories explain how peptides are stabilized in their solid state: the water replacement hypothesis and the glass dynamics hypothesis. The water replacement hypothesis suggests that excipients, such as sugar molecules and polyols, form hydrogen bonds with specific peptide sites, displacing water and preserving the native peptide structure. On the other hand, the glass dynamics hypothesis proposes stabilization through the formation of a rigid, glassy matrix that limits molecular mobility.^8^ Reduced molecular mobility within the glassy matrix results in α and β relaxations, involving gradual translational and rotational motions that significantly affect the diffusion of reactive molecular species. These relaxations, which influence local molecular movements, can be hindered by introducing small molecule plasticizers like poloxamer 407. These plasticizers improve stability by acting as shock absorbers and slowing down degradation.^9^

The swift, one-step spray drying method presents a promising alternative to the time-consuming freeze-drying process for formulating and processing biopharmaceuticals. Unlike a freeze dryer, a spray dryer avoids subjecting the peptide to stresses induced by freezing temperatures allowing for room temperature storage and enhanced stability during handling, storage, transport, and distribution.^10^ The resulting spray-dried peptides are suitable for various administration routes, such as pulmonary, intranasal, and oral, expanding the practical applications of peptide-based therapeutics.^9^ Broadhead et al. investigated the effectiveness of sucrose and HPβCD as stabilizing agents in the spray drying of β galactosidase, a model protein. The solutions underwent processing using a Büchi 190 cocurrent Mini Spray Dryer, maintaining an outlet temperature of 61 ± 2°C. The spray drying approach significantly reduced β galactosidase activity, unaffected by sucrose. However, with HPβCD or a combination of HPβCD and sucrose during spray drying, full catalytic activity was restored upon reconstitution.^11^ These findings highlight the effectiveness of cyclodextrins as stabilizing agents in crafting spray-dried protein pharmaceuticals.

Envisioning the setup of a 3FN involves two separate liquid feeds traversing distinct passages, both undergoing atomization by the gas emerging from a third channel. This configuration allows for the encapsulation of a bioactive within the microcapsule core, resulting in a modified release compared to the blending approach of incorporating the bioactive in the polymer, as observed in the two-fluid nozzle approach.^12^ When a lower viscosity solvent is used as the core medium, it leads to an increased Péclet number,^13^ expediting core formation. Conversely, a low Péclet number contributes to a reduced movement velocity toward the core of the high viscosity medium introduced through the outer nozzle, promoting its accumulation in the shell region of the particles. Recognized for its cost-effectiveness, this method allows for the customization of particle properties.^13^ Our present study involves employing different viscosities for the inner and outer channels of 3FN. The feed solution containing the peptide solution with the HPβCD component is directed into the inner channel, while the high-viscosity feed solution containing sodium alginate and chitosan is conveyed into the outer channel, with varying viscosities resulting in the formulation of the core and shell. Pabari et al. utilized 3FN spray drying process to generate core–shell microcapsules,^12,14^ while Kašpar et al. introduced an *in situ* cross-linking method for chitosan microparticles, demonstrating superior enzymatic activity compared to *ex situ* cross-linking when encapsulating Laccase.^15^ On the other hand, Leena et al. demonstrated the formation of cross-linked core/shell chitosan microparticles for the co-delivery of quercetin and ferulic acid. These studies underscore the effectiveness of the 3FN spray drying technique for core–shell microparticle formation and encapsulation.^12^

Chitosan and alginate, polymers of natural origin, are highly valued in drug delivery due to their proven biocompatibility, biodegradability, and mucoadhesive properties.^16^ Chitosan, derived from chitin, exhibits antimicrobial properties that contribute to enhancing drug stability.^17^ Alginate, derived from brown seaweed, forms cross-linked hydrogel networks that enable controlled and sustained drug release.^18^ HPβCD, a well-established excipient, intervenes to enhance the solubility and stability of drugs with poor water solubility.^19^ It forms inclusion complexes, safeguarding drugs from degradation and improving bioavailability. Poloxamer 407, a thermosensitive gel-forming triblock copolymer, assists with solubility, drug delivery, and formulation stability.^20^

The synergistic effect of Polymyxin B, spray drying, chitosan, alginate, HPβCD, and poloxamer 407 constitutes a holistic approach. Spray drying acts as a protective shield, preventing Polymyxin B from degradation. Chitosan and alginate play crucial roles in ensuring controlled release and targeted delivery.^21^ HPβCD not only improves solubility but also contributes to stability by forming a protective sugar cage that fixes the side branches through hydrogen bonds. Additionally, poloxamer 407^22^ adds to the stability and ensures sustained release. While these components may not directly amplify Polymyxin’s antimicrobial activity, their collective function in refining formulation and delivery indirectly supports the antibiotic’s effectiveness.

Inhalation therapy has emerged as the leading approach for treating respiratory bacterial infections, capitalizing on its heightened effectiveness in overcoming pulmonary biological barriers that traditionally limit the bioavailability of inhaled anti-infectives. Despite significant efforts in the field, current methods often struggle with a persistent increase in bacterial resistance, particularly evident in cases involving opportunistic pathogens like *Pseudomonas aeruginosa*. These infections pose serious threats, especially to immunocompromised patients.^23^ Improving the stability and efficacy of vital antibiotics such as Polymyxin B through multinozzle spray drying processes holds the potential to develop advanced drug delivery systems for combating bacterial infections, particularly those affecting the lungs.

## 2 Materials and Methods

### 2.1 Materials

Chitosan used in this study was characterized as low-molecular-weight, deacetylated chitin (poly(d-glucosamine)), with a high purity, nonanimal derived origin. It has a 99% degree of deacetylation and an average molecular weight (*M*_w_) of 50,000–190,000 Da (Lot# STBH6262). Sodium alginate used consisted of 60–70% mannuronic acid (M) and 30–40% guluronic acid (G), resulting in an M/G ratio of 1.56. It has a degree of polymerization ranging from 400 to 600, and its molecular weight is approximately 30,000–100,000 (Lot# MKBJ2754 V). Hydroxypropyl-β-cyclodextrin (HPβCD) had a molecular weight of approximately 1396 Da, with an average degree of substitution of 0.5–1.3 units of 2-hydroxypropyl (C_3_H_7_O) per glucose unit. Polymyxin B, low-molecular-weight chitosan (50,000–190,000 Da), sodium alginate, HPβCD, acetic acid, poloxamer 407, fluorescein isothiocyanate sodium (FITC), rhodamine B, dialysis sacks (MWCO 12,000 Da), and high-performance liquid chromatography (HPLC) grade acetonitrile were purchased from Sigma-Aldrich (Dorset, U.K.). *Escherichia coli* ATCC 2592 and *P. aeruginosa* NCT10662 were obtained from laboratory stocks held in the microbiology department at the School of Biological Sciences, University of Reading, U.K.

### 2.2 Preparation of Core–Shell Microcapsules by 3FN Spray Drying Technique

In this study, sodium alginate (Alg) and chitosan (CS) were chosen as the wall materials for encapsulating Polymyxin B in two distinct microcapsule forms, utilizing a 3FN spray drying technique. The design parameters, encompassing the polymer ratio, HPβCD and poloxamer 407 amounts, and type of coating polymer, were systematically manipulated using a full factorial design with three center points. The primary focus was on evaluating the responses of yield%, recovery%, and residual moisture. The preparation of the wall material solution involved precise ratios outlined in Table 1. Alg was dissolved in distilled water, while CS was dissolved in acetic acid (1 v/v%). Both solutions underwent meticulous stirring (600 rpm) at 40 °C for 6 h to achieve uniform dissolution, with the incorporation of poloxamer 407 as a stabilizer. This meticulously prepared mixture served as the external phase. The internal phase, comprising Polymyxin B and HPβCD, maintained a consistent amount of 5 mg of Polymyxin B with varying HPβCD amounts based on the factorial design.

**Table 1:** Microparticle Formulations Studied Using Factorial Design, Exploring Different Polymer Types and Amounts, Including HPβCD and Poloxamer 407^a^,^b^

zformulation code	polymer (mg)	HP β CD (mg)	poloxamer 407 (mg)	the used polymer	the residual moisture content %	yield %	recovery %
ALG_L_CD_L_P_L_^PM^	200	10	10	Alg	20 ± 1	70 ± 3	6.7 ± 0.8
ALG_H_CD_L_P_L_^PM^	600	10	10	Alg	20 ± 2	49.5 ± 3.2	4.5 ± 0.2
ALG_L_CD_H_P_L_^PM^	200	50	10	Alg	8 ± 1	63.5 ± 2.5	42.5 ± 0.3
ALG_H_CD_H_P_L_^PM^	600	50	10	Alg	8 ± 1	67.2 ± 1.8	91.8 ± 2.2
ALG_L_CD_L_P_H_^PM^	200	10	50	Alg	13 ± 2	62 ± 2	23 ± 2
ALG_H_CD_L_P_H_^PM^	600	10	50	Alg	20 ± 1	55.5 ± 2.2	10.7 ± 1
ALG_L_CD_H_P_H_^PM^	200	50	50	Alg	20 ± 1	42.3 ± 0.5	8.2 ± 0.8
ALG_H_CD_H_P_H_^PM^	600	50	50	Alg	15 ± 1	45.6 ± 0.7	7.8 ± 0.7
CS_L_CD_L_P_L_^PM^	200	10	10	CS	8 ± 2	61.8 ± 0.8	7.8 ± 1.2
CS_H_CD_L_P_L_^PM^	600	10	10	CS	8 ± 1	46.7 ± 1.2	3.8 ± 0.7
CS_L_CD_H_P_L_^PM^	200	50	10	CS	13 ± 2	68 ± 2	5.6 ± 0.8
CS_H_CD_H_P_L_^PM^	600	50	10	CS	5 ± 1	73 ± 2	5.6 ± 0.2
CS_L_CD_L_P_H_^PM^	200	10	50	CS	14 ± 1	56.3 ± 1.2	5.2 ± 0.2
CS_H_CD_L_P_H_^PM^	600	10	50	CS	9 ± 2	55 ± 1	4.8 ± 0.3
CS_L_CD_H_P_H_^PM^	200	50	50	CS	17 ± 2	59 ± 1	4.7 ± 0.4
CS_H_CD_H_P_H_^PM^	600	50	50	CS	8 ± 1	47.3 ± 0.6	5.5 ± 0.3

a Results of the tested microparticles include the residual moisture content, yield, and recovery percentage.

b Formulation coding key: alginate (ALG), chitosan (CS), HPβCD (CD), poloxamer 407 (P), Polymyxin B (PM), high concentration (H), and low concentration (L).

To comprehensively assess the impact of the studied factors on core–shell formation, formulations were precisely prepared using a laboratory 3FN spray dryer (BUCHI B 290 mini Spray Dryer). The external phase was meticulously delivered through the middle nozzle to facilitate the shell formation. Concurrently, the internal phase was intricately fed through the innermost nozzle, serving as the core bioactive material. The operational parameters were adjusted to guarantee a robust process in the trials with 3FN. The inlet temperature was systematically set at 100 °C, and the outlet temperature was varied to 54 °C. The aspiration rate was fixed at 75%, and the atomizing air flow pressure was maintained at 3 bar. Furthermore, the liquid feed flow rate for the outer fluid (shell) and inner nozzle (core) was held steady at 2 mL/min. The yield was determined by dividing the final weight of the resulting powder by the total weight of the ingredients before the spray drying process.

### 2.3 Viscosity Analysis

The viscosities of the chitosan and alginate solutions (0.4 and 0.1%) were evaluated using a Brookfield viscometer, with measurements conducted using Steady Shear Tests. The viscometer was calibrated with a viscosity standard fluid, ensuring an accuracy of ±1%. Viscosity measurements were expressed in centipoises (cp). The samples were tested with spindle number 1 at a speed of 750 rpm, at a temperature of 50 °C, and for a duration of 12 s.

### 2.4 Recovery Analysis

The quantification of Polymyxin B recovery in polymeric microparticles involved spectrophotometry through high-performance liquid chromatography (Agilent, 1100 Series) with a size exclusion column (Biozen 3 μm dSEC 2200A°, LC column 150 mm × 7.8 mm). The absorbance value of the solution was measured at 298 nm with an injection volume of 10 μL and a flow rate of 1 mL/min. Each formulation, containing precisely 5 mg of Polymyxin B, was dispersed in 2 mL of water and subjected to vortexing for 10 min. Subsequently, the bioactive and polymer matrix were dissolved, resulting in a final concentration of each Polymyxin B at 2.5 mg/mL. Following the incubation period, the actual concentration of Polymyxin B value of the solution was measured. Yield % (EE) was calculated using the formula: Recovery (w/w)% = (Amount of Polymyxin B inside microparticles)/(Theoretical Polymyxin B content).

### 2.5 Thermogravimetric Analysis

Thermogravimetric analysis (TGA) was carried out using TGA Q50. The procedure involved weighing around 10 mg of the samples, followed by heating them from ambient temperature at a rate of 10 °C/min. The TA Universal Analysis software was then employed to characterize thermal events.

### 2.6 Assessment of Microparticle Morphology

To validate the formation of core/shell structures, two distinct fluorescent dyes, namely, fluorescein isothiocyanate (FITC) with an excitation wavelength of 494 nm and emission at 520 nm and rhodamine B with an excitation wavelength of 540 nm and emission at 625 nm, were employed in the core and shell solutions, respectively. The distribution of fluorescent dyes within the microparticles was scrutinized using a fluorescence microscope (AMG EVOS Fl AMF 4301 Fluorescence Microscope).

### 2.7 Focused Beam Reflectance Measurement (FBRM) for Polymeric Microparticles Size Analysis

The FBRM probe (Model P1 8/91, Mettler Toledo Lasentec, D600T, Switzerland) was positioned within an ethanol-filled suspension vessel containing polymeric microparticles. The FBRM D600T employed a laser light beam rotating at a constant speed of 2 m/s as its light source. This process involved capturing reflected laser energy through backscatter from particles adjacent to the sapphire window orifice. Microparticles (10 mg) were introduced to a beaker containing 20 mL of ethanol, with continuous stirring facilitated by a BioCote hot plate stirrer (500 rpm). The measurements were conducted over a 60 min duration, with data collected at 1 min intervals. The specific placement between the propeller stirrer and the inner side of the suspension vessel was strategically chosen to ensure optimal turbulence. This positioning facilitated the measurement of a representative sample of the particle system. The calibrated FBRM D600T was utilized to assess particles within the 1 to 1000 μm range. Throughout this duration, systematic data collection took place, focusing on obtaining precise and sensitive chord length distributions (CLD) and counting each size at various time points. Specifically, histograms were generated for particle size at a 15 min time point to capture the diameter characteristics of the polymeric microparticles.

### 2.8 Interaction between the Core and Wall Material

Fourier transform infrared (FTIR) spectroscopy (PerkinElmer Spectrum One Waltham, MA) with the attenuated total reflection (ATR) mode was employed to examine potential interactions between loaded Polymyxin B and the materials utilized in the formulation of microparticles. Spectra were recorded within the range of 650–4000 cm^–1^ at a resolution of 4 cm^–1^, with a minimum of 16 scans.

### 2.9 Assessment of the Physical State of Bioactive Compounds in Microparticles

The X-ray diffraction (XRD) pattern of microparticles was examined through X-ray powder diffraction (XRPD). A Bruker D8 advance X-ray diffractometer (Bruker AXS GmbH, Germany) with a Cu source, θ–θ geometry, and a Lynx eye position sensitive detector was employed for scanning. The diffractometer was operated at a generator voltage of 40 kV and a generator current of 40 mA. Analysis was carried out using DFFRAC plus XRD commander software (Bruker AXS GmbH, Germany), covering a 2θ range of 5–45°, with a step size of 0.02°, and a time per step of 1.33 s.

### 2.10 *In Vitro* Release Profile

*In vitro* release of Polymyxin B from core–shell microcapsules was conducted using the dialysis bag method. To assess the cumulative release of the bioactive from the encapsulated matrix, 10 mg of spray-dried microparticles was dispersed in 5 mL of deionized water by vortexing for 3 min. In this study, deionized water was chosen as the medium for the release study. This approach ensures a clearer analysis and interpretation of results, free from the potential confounding effects of complex medium compositions.^57,58^ The resulting mixture was then transferred to a dialysis bag with a 12 kDa molecular weight cutoff range. The release of Polymyxin B took place under sink conditions in 50 mL of release medium at 37 °C, with agitation using a BioCote hot plate stirrer at 100 rpm. Cumulative release was monitored for up to 24 h. At various time intervals, 1 mL of the release medium was withdrawn and replaced with an equal amount of fresh release medium to maintain a constant volume. The quantification of Polymyxin B in the release medium was then performed against standard graphs. Deionized water offers a simpler and more controlled environment, minimizing variables that could interfere with the isolation process. This approach ensures a clearer analysis and interpretation of results, free from the potential confounding effects of complex medium compositions.

The release profiles of Polymyxin B were analyzed using different kinetic models, including zero-order kinetics, first-order kinetics, the Higuchi model, the Korsmeyer–Peppas equation, the Hixson–Crowell cube root law, and the Peppas–Sahlin model, to characterize the mechanism of bioactive molecule release. The constants of release kinetics and the regression coefficients (*R*^2^) were calculated from the slope of plots by linear regression analysis. Moreover, the examination of the release behavior was tested for rhodamine B in the shell and FITC in the core using a microplate reader (FlexStation 3 Multi-Mode Microplate Reader), with the intention of validating encapsulation achieved through using a 3FN spray dryer.

### 2.11 Antimicrobial Formulation Testing Methodology for MIC, MBC, and Controls

From single colonies, *E. coli* and *P. aeruginosa* were cultured in 2 mL of Lysogeny broth (LB) for 18 h, with resulting cultures diluted to an OD_600_ of 1. Serial dilutions were performed using pure Polymyxin B and the tested formulations (ALG_L_CD_H_P_L_^PM^, ALG_H_CD_H_P_L_^PM^, CS_L_CD_H_P_L_^PM^, and CS_H_CD_H_P_L_^PM^), ranging from 0.39 to 500 μg/mL calculated according to the final amount of Polymyxin B in each studied formulation. These were diluted with sterile Mueller Hinton broth (MHB) before inoculation with the target bacteria. A volume of 10 μL of bacterial suspension containing 10^6^ colony forming units (CFUs) per milliliter was added to each well in 200 μL volumes in flat-bottomed 96-well plates (Greiner).

OD_600_ readings were recorded every 60 min at 37 °C, using a Stratus microplate reader and shaking overnight at 37 °C under agitation at 150 rev/min, on an orbital shaker for 18 h. Following the experimental phase, the OD_600_ data were extracted and subjected to detailed analysis, ensuring a comprehensive assessment of the experimental outcomes. The MIC was identified as the lowest bacteriostatic concentration of the formulation inhibiting visible growth of the bacteria.

Control wells were used to test the excipients present in various formulations, testing for the respective antimicrobial effects. Wells solely containing MHB (i.e., without any antimicrobial formulation or antibiotics) were inoculated with bacteria, providing a baseline of unimpeded growth for comparison. Separate wells without bacteria were treated with the highest tested concentration (500 μg/mL) of the formulations and excipients, assessing intrinsic contamination in the absence of the tested bacteria. Following MIC determination, 10 μL aliquots from test wells exhibiting no visible growth were transferred onto fresh Mueller Hinton agar plates and incubated for 24 h at 37 °C. The MBC was identified as the lowest concentration with no bacterial growth (i.e., no colony formation) on agar plates.

Utilizing FITC as the core label and Rhodamine B as the shell label, the study investigated the dynamic fluorescence response following the treatment of *E. coli* and *P. aeruginosa* after 1 and 18 h of incubation. Additionally, a fluorescence signal was observed under a fluorescence microscope (AMG EVOS Fl AMF 4301 Fluorescence Microscope).

### 2.12 Biofilm Formation

For biofilm formation studies, after the designated incubation period (typically 24 h), the 96-well plate was inverted over a discard container to remove the media and any unattached cells. This step was followed by a series of rinses in sterile water to remove residual media and nonadherent cells. A volume of Crystal violet solution equivalent to 200 μL was added to each well, ensuring complete coverage, and the plate was then incubated at room temperature (25 °C) for 20 min to allow for sufficient staining of the biofilm. Following the incubation with Crystal violet, the wells were gently rinsed with sterile water to remove excess stain. The plate was then air-dried at room temperature under gentle airflow to facilitate complete drying of the wells. For qualitative evaluation, images of the dried wells were taken by using a digital camera equipped with appropriate resolution and focusing capabilities.

To quantify biofilm formation, 125 μL of 30% acetic acid in water was added to each well to dissolve the crystal violet bound to the polypropylene wall. Following incubation, the dissolved crystal violet was transferred to a new 96-well plate, and absorbance at 550 nm was measured using a plate reader (FlexStation 3 Multi-Mode Microplate Reader). A blank containing 30% acetic acid in water was utilized for calibration.

### 2.13 Statistical Analysis

All experiments were conducted in triplicate, and data analysis employed analysis of variance (one-way ANOVA) using Prism version 9.4 to determine the statistical significance (*P* < 0.05).

## 3 Results and Discussion

### 3.1 Preparation of Core–Shell Microcapsules by 3FN Spray Drying Technique

In this study, we prepared core–shell microcapsules of Polymyxin B using a 3FN spray drying method. The flow rates of the core and shell solutions underwent optimization through preliminary investigations, leading to the selection of 2 mL/min for the shell and the core fluid, respectively. The optimized conditions for the formation of core–shell microparticles and the studied responses, the percentage of residual moisture content, yield, and recovery%, are listed in Table 1.

### 3.2 Viscosity Analysis

For alginate, the viscosities at high (0.4% w/v) and low (0.1% w/v) concentrations were 44.50 and 14.50 cP, respectively, as measured using the Brookfield viscometer. For chitosan, the viscosities at high (0.4% w/v) and low (0.1% w/v) concentrations were 29.7 and 6.9 cP, respectively.

### 3.3 Yield and Recovery Analysis

Polymyxin B underwent microencapsulation within a sugar cage (HPβCD) and alginate sodium or chitosan LW blend polymers using the conventional 3FN spray drying process. In-depth calculations of recovery and yield percentages for each formulation revealed that ALG_H_CD_H_P_L_^PM^ and ALG_L_CD_H_P_L_^PM^ (91.76 and 42.49, 67.20 and 63.50%, respectively) performed exceptionally well in alginate-based formulations. On the other hand, CS_L_CD_H_P_L_^PM^ and CS_H_CD_H_P_L_^PM^ (5.57 and 5.59, 68.10 and 73.40%, respectively) excelled in chitosan-based formulations. This indicates a significant around 16-fold rise in Polymyxin B recovery with alginate in comparison to chitosan microparticles, attributed to the higher recovery in each group. Nevertheless, a decrease in the recovery percentage of Polymyxin B was observed with decreasing polymer or HPβCD ratios or increasing poloxamer 407 ratio in the examined formulations as listed in Table 1.

In the 3FN spray drying process, the simultaneous introduction of two different fluids in viscosity through the innermost and middle nozzles significantly influenced the recovery % of the core component, which increased with the increasing viscosity differences between the two solutions (shell and core). In accordance with the findings of Pabari et al., there was an enhanced yield % in the fluid nozzle process due to viscosity disparities between the core (with low viscosity) and the shell (with high viscosity) feed solutions.^14,24^ A parallel effect was observed by França et al. in the 3FN spray drying process for the preparation of chitosan core and cross-linked chitosan shell.^25^ In our study, the interdiffusion phenomenon resulted in a marked increase in the recovery % of the core bioactive molecule (Polymyxin B) within the high alginate concentration formulation (ALG_H_CD_H_P_L_^PM^). Furthermore, Polymyxin B, a cationic polypeptide antibiotic, is expected to be encapsulated more efficiently in alginate microparticles with a negative surface charge than in a chitosan matrix with a positive surface charge.^26^

### 3.4 Thermogravimetric Analysis

TG analysis revealed a weight loss in the temperature range of 30 to 150 °C for each prepared formulation, indicating the presence of residual humidity and water of hydration within the microparticles post formulation using the 3FN spray dryer. The results in Table 1 show residual humidity percentages of 8, 8, 13, and 5% for ALG_H_CD_H_P_L_^PM^, ALG_L_CD_H_P_L_^PM^, CS_L_CD_H_P_L_^PM^, and CS_H_CD_H_P_L_^PM^, respectively. Notably, it is observed that the higher the poloxamer 407 content, the higher is the residual moisture content, possibly linked to the high hygroscopic properties of poloxamer 407.

The hygroscopic nature of poloxamer 407 leads to higher residual humidity in the final product. The 3FN spray drying process balances the drying rate and particle quality; further moisture reduction could compromise particle integrity. Additionally, water is bound within the microparticles as hydration water and in semicrystalline polymers, making it difficult to remove without affecting their structure.^6,12^ Lowering the residual humidity further could result in brittle and unstable particles. The current 8% residual humidity poses no risk of microbial contamination due to the antimicrobial agent polymyxin. Therefore, maintaining current humidity levels is essential for the quality and stability of the microparticles.^20,27^

### 3.5 Morphology of Prepared Microparticles

To confirm the presence of core/shell structures, two different fluorescent dyes were used: fluorescein isothiocyanate (FITC) in the core solution and rhodamine B in the shell. In formulations containing chitosan, fluorescence from both FITC and rhodamine B was detected within the microparticle structures.^28^

**Figure 1:**
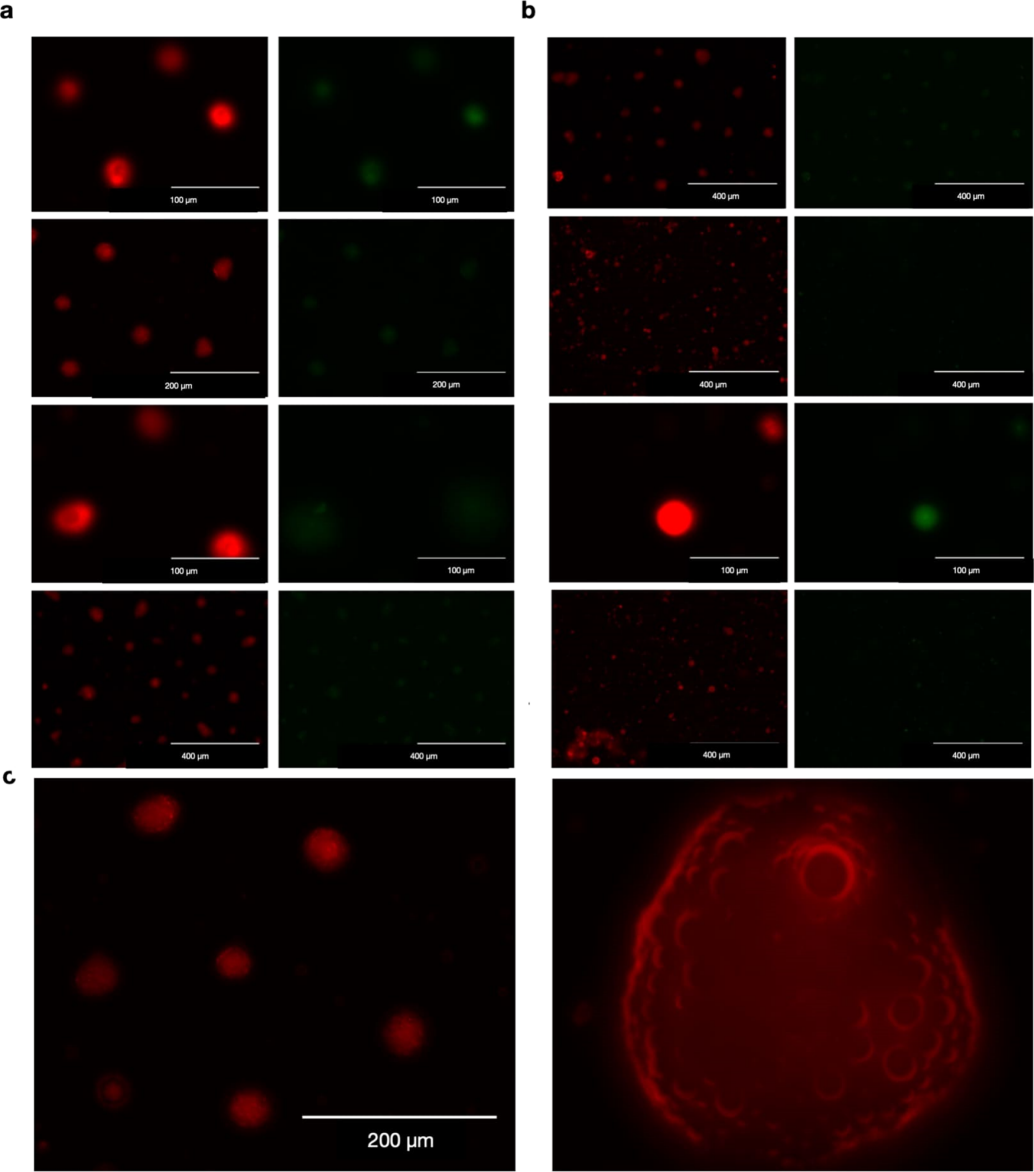
Microscopic examination reveals variations in fluorescence intensity within the core/shell layers. (a) Notable fluorescence patterns in ALG_H_CD_H_P_L_^PM^ showcasing distinct core/shell layers. (b) Fluorescence microscope visualization depicting the reduction of the core component in ALG_L_CD_H_P_L_^PM^. (c) Fluorescence microscopic image revealing the porous structure and morphology of the microparticles.

Microparticles prepared with a high ratio of high-viscosity alginate (ALG_H_CD_H_P_L_^PM^) exhibited distinct layers of green fluorescence from the core and red fluorescence from the shell, forming a ring-like morphology (Figure 1a). Using a low-viscosity aqueous solution as the core solvent in the 3FN spray process increased the Péclet number, leading to rapid crust formation. The higher Péclet number reduced the diffusion velocity of the solute through the core nozzle, resulting in its enrichment in the core of the particles rather than in the shell. This accelerated crust formation protected the core Polymyxin B from processing temperatures during the spray drying procedure and effectively controlled the release rate.^9^

As shown in Figure 1b using the fluorescence microscope, the signal of the core component (FITC) significantly diminished in the core with a lower alginate concentration in ALG_L_CD_H_P_L_^PM^. In contrast, polymer concentration in chitosan microparticles had no significant impact. The reduced wall thickness, presence of FITC in the shell, and increased core-to-wall ratio in the chitosan (CS) formulations led to lighter differentiation in fluorescence between the two filters.^12^

The choice of core and shell materials and variations in formulation viscosity significantly affect the morphology and fluorescence properties of the prepared microparticles. These findings emphasize the importance of controlling formulation parameters to achieve the desired core/shell structures and functionality in microparticle drug delivery systems.^9^

The Péclet number plays a crucial role in describing how substances move within core–shell microcapsules, balancing advection (fluid flow) and diffusion. A higher Péclet number suggests that advection dominates, meaning the substance moves faster with the fluid flow than it diffuses through the medium.^13^ Our study suggested a higher Péclet number in the core, which has a low-viscosity internal phase. This means substances in the core experience faster transport, mainly due to the fluid movement. On the other hand, the shell, made up of a high-viscosity external phase, has a lower Péclet number. This indicates that diffusion has a more significant impact on substance movement in the shell, with less reliance on fluid.^13^ The fluorescence microscopic image reveals the porous structure and surface morphology of the microparticles (Figure 1c).

The impact of the porous structure on peptide stability was evaluated in relation to its effect on the storage conditions. While the increased surface area of porous particles can enhance drug release rates and improve interactions with target sites, potentially leading to more effective therapeutic outcomes, it can also expose the peptide to environmental factors such as humidity and oxygen, which may affect its stability.^1^ Measures have been taken to mitigate these risks, including optimizing the encapsulation efficiency and controlling residual moisture levels through the spray drying process. The residual humidity in our formulations ranges from 5 to 13%, which is managed to minimize degradation.^29^ Although the antimicrobial properties of polymyxin contribute to mitigating microbial contamination risks, they do not fully ensure stability in porous structures. To further protect the particles, proper packaging under controlled conditions, such as low humidity and nitrogen flushing, is employed.^6,30^

To confirm the presence of core/shell structures, we utilized two different fluorescent dyes: fluorescein isothiocyanate (FITC) in the core solution and rhodamine B in the shell.^31^ In formulations containing chitosan, fluorescence from both FITC and rhodamine dyes could be detected within the microparticle structures.

Conversely, microparticles prepared through a high ratio of alginate with high viscosity (ALG_H_CD_H_P_L_^PM^) exhibited fluorescence in green (from the core) and red (in the shell) as distinct layers, forming a ring-like morphology (Figure 1a). This was due to the use of a low-viscosity aqueous solution as a core solvent in the 3FN spray process, contributing to an increased Péclet number and rapid crust formation.^32^ The higher Péclet number reduced the diffusional velocity of the solute fed through the core nozzle, resulting in its enrichment in the core of the particles rather than in the shell. Consequently, the accelerated crust formation played a pivotal role in protecting the core Polymyxin B from processing temperature during the spray drying procedure and effectively controlling the release rate.^9^

The fluorescence microscopic image in Figure 1b shows that the signal of the core component (FITC) notably diminished in the core with a lower alginate concentration in ALG_L_CD_H_P_L_^PM^. Conversely, the polymer concentration in chitosan microparticles showed no significant impact. The lighter differentiation in fluorescence between two filters in the chitosan formulation resulted from a reduced wall thickness along with the presence of FITC in the shell and an increase in the core-to-wall ratio.^33^

The Péclet number plays a crucial role in describing how substances move within core–shell microcapsules, balancing advection (fluid flow) and diffusion. Our study suggested a higher Péclet number in the core, which has a low-viscosity internal phase.^13^ This means substances in the core experience faster transport, mainly due to the fluid movement. On the other hand, the shell, made up of a high-viscosity external phase, has a lower Péclet number. This indicates that diffusion has a more significant impact on substance movement in the shell, with less reliance on fluid.^13^ The fluorescence microscopic image depicts the porous structure and surface morphology of the microparticles.

### 3.6 Focused Beam Reflectance Measurement for Polymeric Microparticle Size Analysis

**Figure 2:**
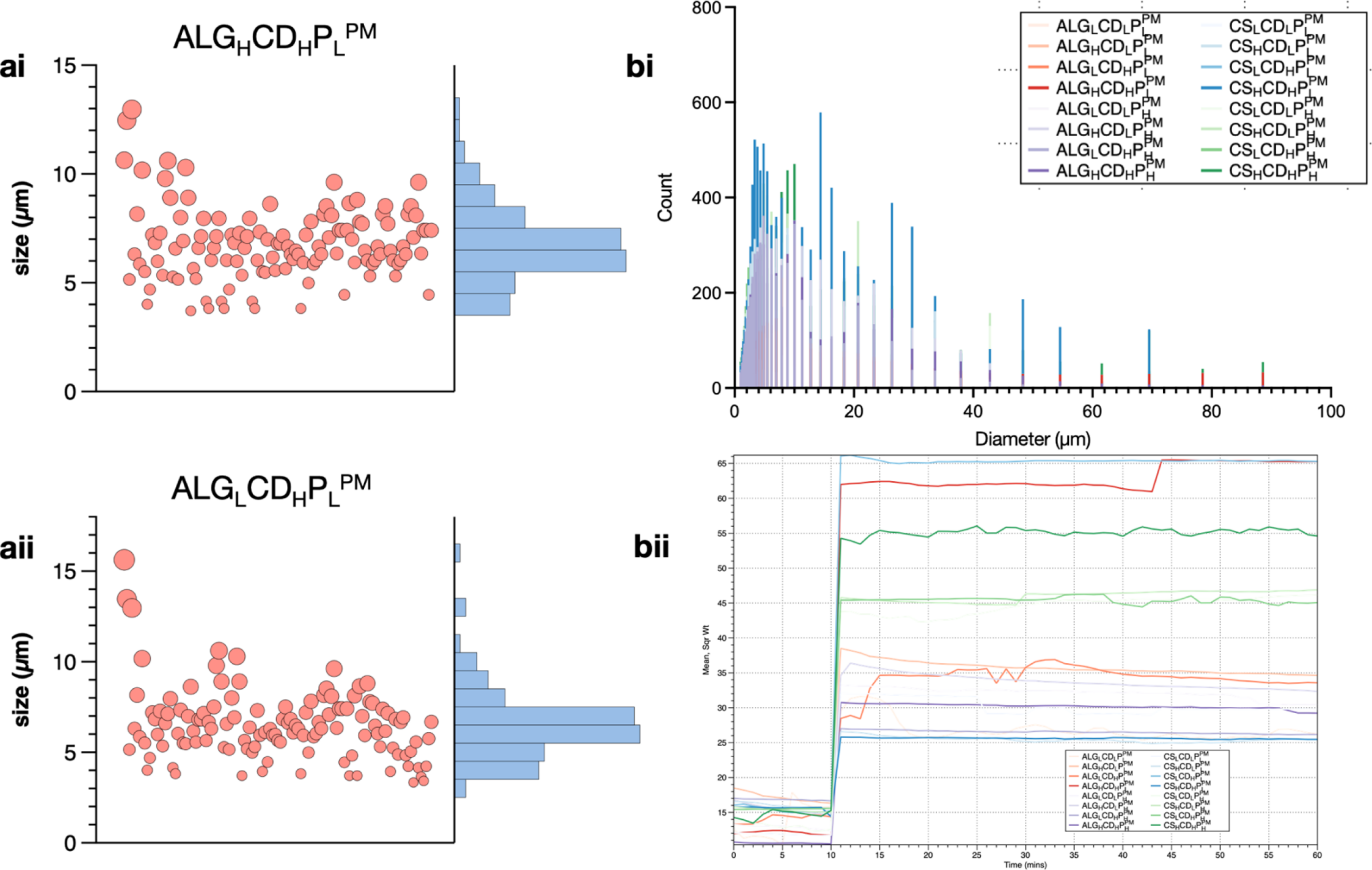
Size distribution of microencapsulated Polymyxin B formulations. (ai, aii) Size distribution histograms for ALG_H_CD_H_P_L_^PM^ and ALG_L_CD_H_P_L_^PM^, respectively. (bi) Real-time focused beam reflectance measurement (FBRM) analysis indicating a chord length distribution below 10 μm. (bii) Slight increase in the count of particles ranging from 5 to 15 μm observed in ALG_H_CD_H_PL^PM^ over 1 hour.

The analysis of the size distribution histograms reveals that the mean and median sizes were 6.7 ± 1.2 and 6.5 ± 1.5 μm for ALG_H_CD_H_P_L_^PM^ (Figure 2ai) and 6.5 ± 0.7 and 6.3 ± 0.8 μm for ALG_L_CD_H_P_L_^PM^ (Figure 2aii), respectively.^34^ During the real-time particle size analysis using the FBRM technique, which measures chord length across the 1 to 1000 μm (μm) range within the 60 min interval, a consistent observation is that the square weighted median of the chord length remains below 10 μm, at the 15 min point.^31^ The correlation between the mean particle size, evaluated through microscopic examination and FBRM results, underscores the effectiveness of utilizing the square weighted mean in the chord length distribution for estimating particle diameter as illustrated in Figure 2bi. Even after 1 hour, the maximum particle count continues to concentrate within the 5–15 μm size range. This phenomenon is linked to the low solubility of chitosan and alginate, the shell materials in our formulation in ethanol. While the overall particle size for ALG_H_CD_H_P_L_^PM^ remains stable, a slight increase in the 5–15 μm count is observed, potentially attributable to a granulation mechanism,^35,36^ as depicted in Figure 2bii. D90 particle sizes were 6.7 ± 1.2, 6.5 ± 1.5, and 8.3 ± 1.2 μm for ALG_H_CD_H_P_L_^PM^ (Figure 2ai) and 6.5 ± 0.7, 6.3 ± 0.8, and 7.4 ± 0.7 μm for ALG_L_CD_H_P_L_^PM^.

For effective lung delivery, particles typically need to be within the 1–5 μm range for deep lung penetration, while particles larger than 5 μm generally deposit in the upper airways and those smaller than 1 μm may be exhaled before deposition.^37,38^ Despite our formulation predominantly producing particles in the 5–15 μm range, these particles remain highly promising for targeted delivery. The 5–15 μm range is advantageous for upper airway deposition, beneficial in treating localized respiratory conditions such as bronchitis and COPD, where higher local drug concentrations can enhance therapeutic outcomes.^39,40^ Furthermore, the particle size is ideal for delivery via nebulizers or dry powder inhalers, which are critical in respiratory therapy.^40^

Beyond respiratory applications, these microparticles are versatile for nonrespiratory uses. In wound care, the 5–15 μm particles can be integrated into topical formulations like creams or dressings, providing sustained release and high local drug concentrations to effectively treat skin infections while minimizing systemic exposure.^41^ Additionally, these particles are suitable for intravesical therapy, delivering drugs directly to the bladder to treat urinary tract infections, ensuring localized high concentrations and reducing systemic toxicity.^33,34^ Thus, while the particle size may not align with traditional parameters for deep lung delivery, their potential across various medical applications—such as respiratory therapy, wound care, and intravesical therapy—underscores the versatility and promise of these formulations.^37,39,41^

### 3.7 Interaction between the Core and Wall Material

**Figure 3:**
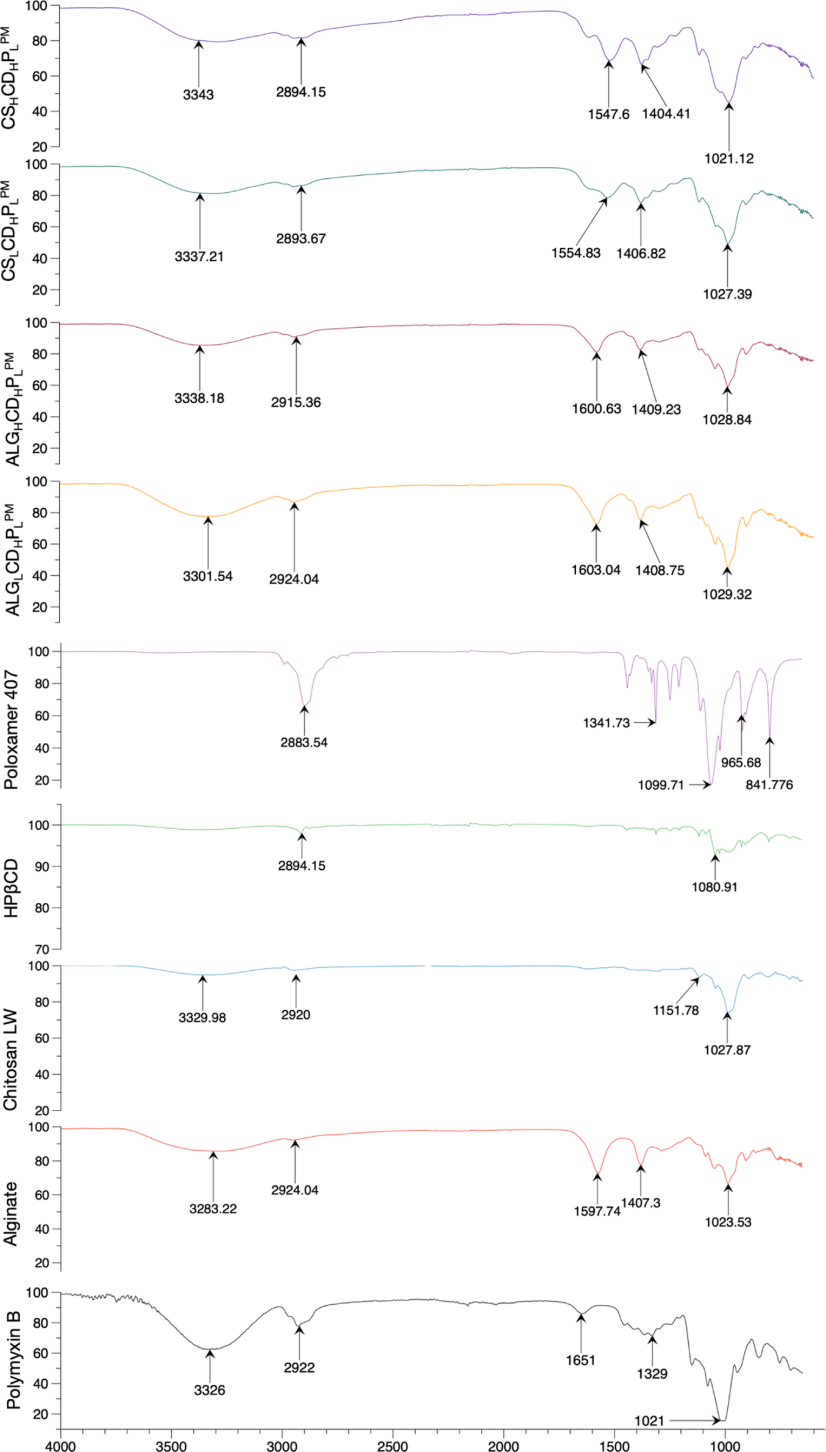
FTIR spectral analyses. Spectroscopic insights of FTIR spectrum unravelling intricate signals and molecular interactions between Polymyxin B and the components within alginate or chitosan microparticles.

The FTIR spectral analysis of polymyxin, alginate, chitosan, cyclodextrin, and poloxamer, and their formulations with varying concentrations of alginate and chitosan in Figure 3, revealed detailed changes in hydrogen-bonding and electrostatic interactions, especially between the functional groups present in these compounds. For polymyxin, the original peaks at 1021 cm^–1^ (C–O stretching), 1329 cm^–1^ (N–H bending/C–N stretching), 1651 cm^–1^ (amide I, C=O stretching in proteins/peptides), 2922 cm^–1^ (C–H stretching), and 3326 cm^–1^ (N–H or O–H stretching) shifted in the formulations, indicating altered interaction environments. In low concentration alginate formulations, the shifts to 1029 cm^–1^ (C–O stretching), 1408 cm^–1^ (symmetric stretching of carboxylate, COO^–^),^40^ 1603 cm^–1^ (asymmetric stretching of carboxylate, COO^–^), 2924 cm^–1^ (C–H stretching), and 3301 cm^–1^ (O–H stretching) suggest enhanced electrostatic interactions between the negatively charged carboxylate (COO^–^) groups of alginate and the positively charged ammonium groups of polymyxin. Additionally, changes in the O–H stretching region from alginate’s original peak 3283 to 3301 cm^–1^ indicate alterations in hydrogen bonding. High concentration alginate formulations show even more pronounced shifts to 1028 cm^–1^ (C–O stretching), 1409 cm^–1^ (symmetric stretching of carboxylate, COO^–^), 1600 cm^–1^ (asymmetric stretching of carboxylate, COO^–^), 2915 cm^–1^ (C–H stretching), and 3338 cm^–1^ (O–H stretching), reflecting even stronger electrostatic interactions and more extensive hydrogen-bonding networks due to the increased alginate content.^42,43^ For chitosan, the FTIR peaks shifted to 1027 cm^–1^ (C–O–C stretching), 1406 cm^–1^ (symmetric stretching of carboxylate, COO^–^), 1554 cm^–1^ (amine bending), 2893 cm^–1^ (C–H stretching), and 3337.21 cm^–1^ (N–H or O–H stretching) in low concentration formulations, and to 1021 cm^–1^ (C–O–C stretching), 1404 cm^–1^ (symmetric stretching of carboxylate, COO^–^), 1547 cm^–1^ (amine bending), 2894 cm^–1^ (C–H stretching), and 3343 cm^–1^ (N–H or O–H stretching) in high concentration formulations.^1,44,45^ These shifts highlight primarily hydrogen-bonding interactions between chitosan and polymyxin as there are minimal electrostatic interactions due to the lack of significant charge complementarity between chitosan and polymyxin. The O–H stretching region in chitosan shifted from its original peak 3329 to 3337.21 cm^–1^ (low concentration) and 3343 cm^–1^ (high concentration), indicating stronger hydrogen bonding at higher chitosan concentrations. Additionally, the O–H stretching region in alginate shifted from 3283 to 3301 cm^–1^ (low concentration) and 3338 cm^–1^ (high concentration), reflecting stronger hydrogen bonding at higher concentrations. Overall, the FTIR analysis demonstrates that higher concentrations of alginate and chitosan improve hydrogen bonding and, in the case of alginate, electrostatic interactions with polymyxin.^46^ These interactions significantly impact the stability and functionality of the formulations, with stronger hydrogen bonds and electrostatic bonds in high concentration formulations leading to more stable and potentially more effective formulations.^40,47^

### 3.8 Assessment of the Physical State of Bioactive Compounds in Microparticles

**Figure 4:**
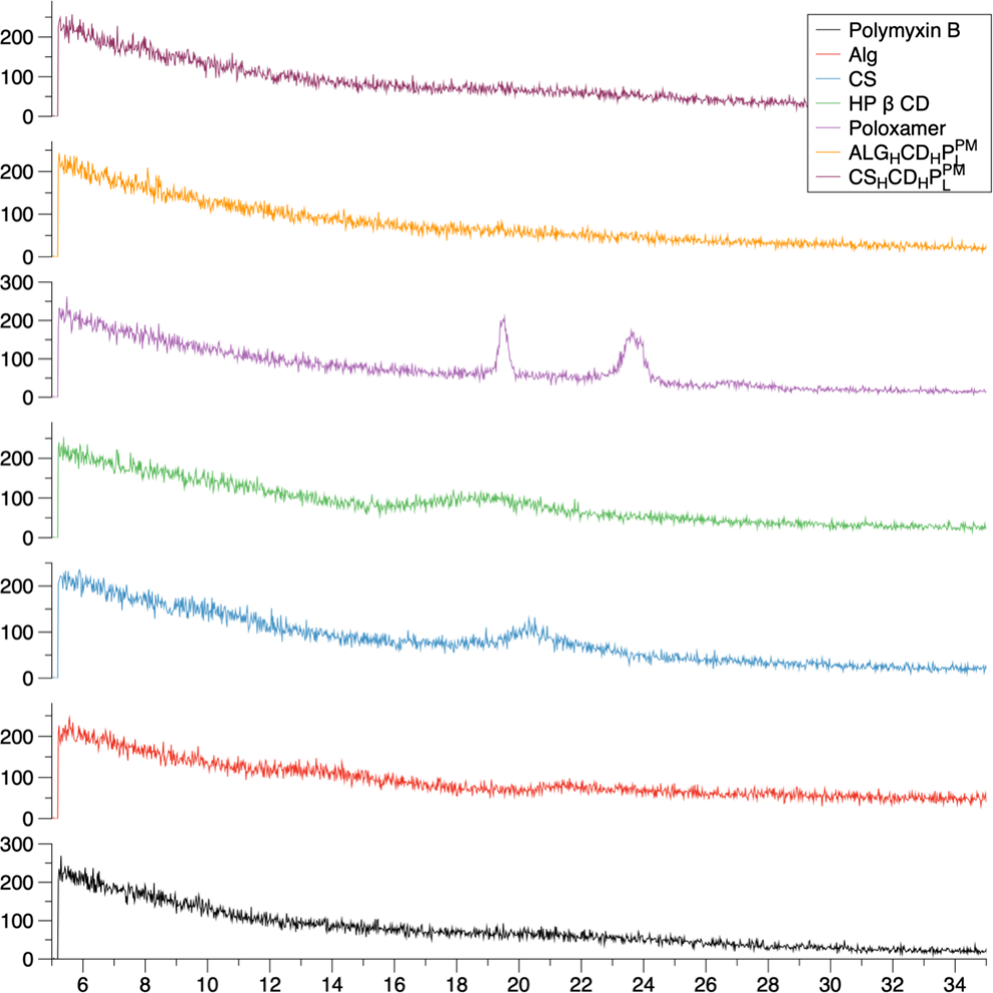
XRD analyses. Crystalline transformation: X-ray diffraction reveals the influence of microencapsulation on the crystallinity of Polymyxin B, emphasizing its persistent amorphous nature.

Figure 4 shows that the impact of microencapsulation on the crystallinity of Polymyxin B was assessed by analyzing the X-ray diffraction pattern. Chitosan and HPβCD, employed in microparticle preparation, exhibited two distinct broad peaks at 20 and 18°, while poloxamer 407 exhibited slightly sharp peaks at 20 and 22.5°. However, upon encapsulation, characteristic diffraction peaks of these ingredients were not observed, signifying their complete transition to an amorphous state. Microparticles ALG_H_CD_H_P_L_^PM^ and CS_H_CD_H_P_L_^PM^, produced through the spray drying process, exhibited no peaks, emphasizing the amorphous nature of the resulting matrix.^48^

Littringer et al. also noted similar findings in their study, where needle-like crystalline trans-resveratrol was transformed into the amorphous form upon encapsulation in hydroxypropyl methylcellulose.^28^ This transformation led to a notable enhancement in the solubility of resveratrol in both deionized water and a PBS solution. Additionally, encapsulation increased the effective surface area of bioactive compounds dispersed in microparticles, facilitating rapid dissolution of the water-soluble matrix. Consequently, this enhanced the absorption of encapsulated molecules, ultimately improving their bioavailability.^19^ In our investigation, the outcomes of the LC-MS testing yielded inconclusive results. While pure Polymyxin B was successfully identified using LC-MS, its absence in the microparticle analyses presents a significant discrepancy that requires examination.

One possible explanation for this inconsistency lies in the well-documented challenge of ionization suppression, commonly encountered in mass spectrometry analyses. In the context of our formulation analyses, it is conceivable that the Polymyxin B molecules remained trapped within the microparticle structures, which contain a high ratio of high-molecular-weight long-chain polymers, such as alginate. This confinement could have hindered their release into the solvent and subsequent ionization, resulting in their undetectability despite their presence in the sample.^23,33^ Additionally, the presence of other components within the microparticles, such as HPβCD and poloxamer 407 in high ratios, may have further contributed to ionization suppression. These components could have interfered with the ionization process of Polymyxin B, exacerbating the challenge of its detection.^49^ Stability assessment via HPLC with a size exclusion column reveals consistent retention times (4.2 min) before and after formulation, demonstrating the peptide stability post encapsulation. This solidifies the evidence of peptide stability following formulation.^50^

### 3.9 *In Vitro* Release Profile

**Figure 5:**
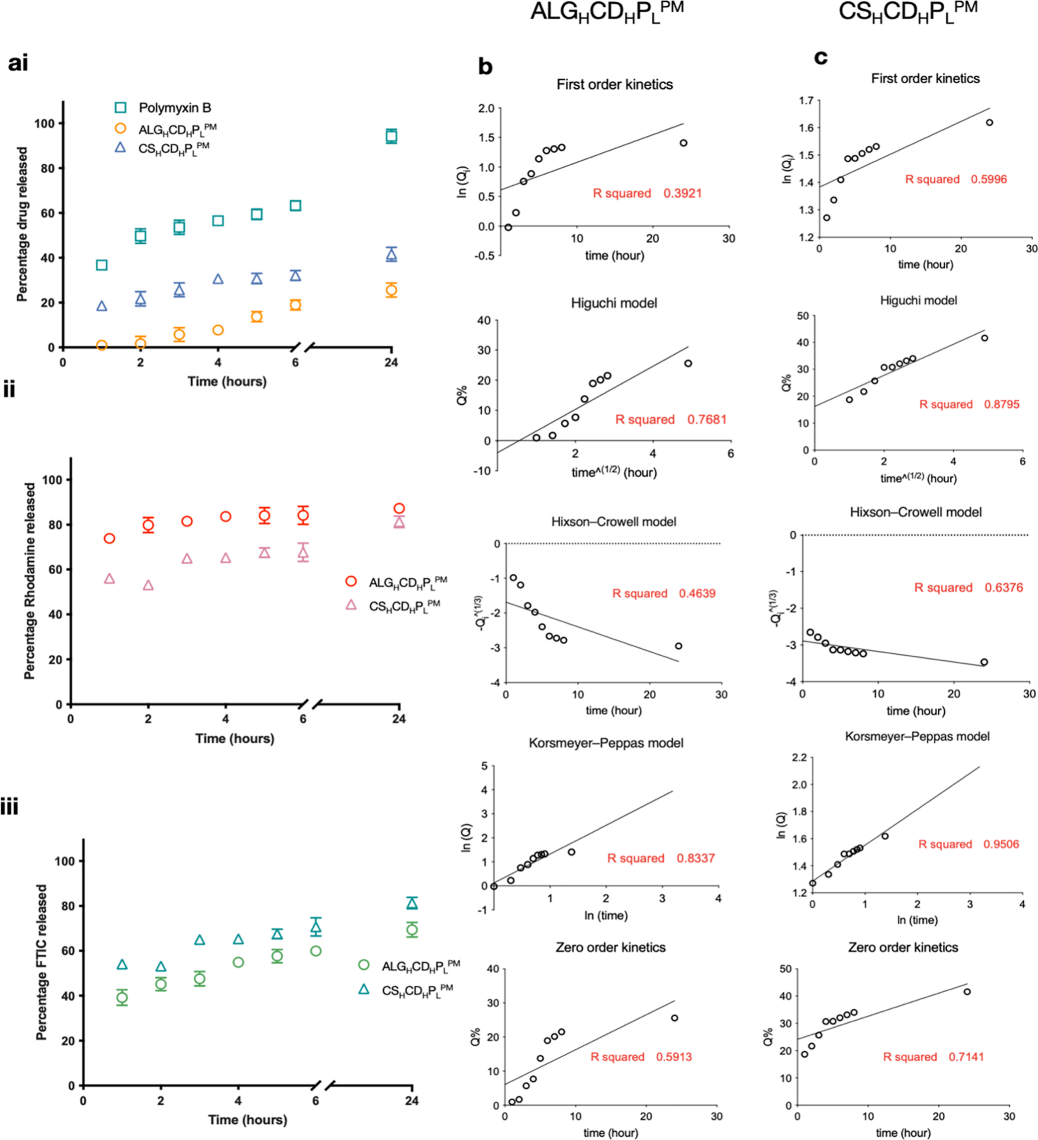
Polymyxin B release kinetics. (ai) Cumulative release profiles of Polymyxin B from ALG_H_CD_H_P_L_^PM^, CS_H_CD_H_P_L_^PM^, and free solution over 24 h. (aii,iii) Controlled release patterns of rhodamine and FITC from ALG_H_CD_H_P_L_^PM^ and CS_H_CD_H_P_L_^PM^, respectively, highlighting core–shell microparticles’ potential for immediate and sustained effects. (b) Comparative analysis of the release mechanisms.

Figure 5ai illustrates the release patterns of Polymyxin B from microparticles. At the 24 h time point, ALG_H_CD_H_P_L_^PM^ and CS_H_CD_H_P_L_^PM^exhibited cumulative releases of around 25 ± 3 and 41.6 ± 2.8%, respectively. Notably, CS_H_CD_H_P_L_^PM^ demonstrated a faster Polymyxin B release, attributed to its weaker structure compared to ALG_H_CD_H_P_L_^PM^. This underscores the effective control exerted by core–shell microparticles (ALG_H_CD_H_P_L_^PM^) over Polymyxin B release compared to the free solution, which released 94.2 ± 2.5% in the same time frame. The free solution showed a notable high release of Polymyxin B within the first hour, reaching 36.7 ± 2.3%, surpassing the release from ALG_H_CD_H_P_L_^PM^ and CS_H_CD_H_P_L_^PM^ in 24 h and 6 h, respectively.

The release of fluorescence materials was also investigated. ALG_H_CD_H_P_L_^PM^ released 39.2 ± 3.5% of FITC and 73.8 ± 0.5% of rhodamine within 24 h, while CS_H_CD_H_P_L_^PM^ released 54.2 ± 1.3% of FITC and 56.2 ± 0.5% of rhodamine. In ALG_H_CD_H_P_L_^PM^, FITC from the core exhibited controlled release with a faster release of rhodamine in the shell. Despite using the same polymer concentration in ALG_H_CD_H_P_L_^PM^ and CS_H_CD_H_P_L_^PM^, rhodamine release from the wall was faster in ALG_H_CD_H_P_L_^PM^, while the opposite was observed for FITC in the core. This was attributed to a thinner wall in CS_H_CD_H_P_L_^PM^ due to the interdiffusion of the polymer solution within the core, increasing the core-to-wall ratio. Thus, the optimized ALG_H_CD_H_P_L_^PM^ co-delivery microparticles have the potential for immediate effects with faster release of shell material and sustained release of core material as shown in Figure 5aii,iii.

To understand the release mechanism, Polymyxin B release profiles from ALG_H_CD_H_P_L_^PM^ and CS_H_CD_H_P_L_^PM^ were fitted to various kinetic models. The Korsmeyer–Peppas model indicated diffusion and dissolution control, with pseudo-Fickian diffusional mechanism for Polymyxin B and anomalous transport.^51,52^ The Peppas model confirmed diffusion dominance over polymer relaxation or swelling, with ALG_H_CD_H_P_L_^PM^ matrices exhibiting a swelling-assisted diffusion-controlled mechanism as shown in Figure 5b.

Our study demonstrates that formulations with high ratios of HPβCD and sodium alginate (ALG_H_CD_H_P_L_^PM^) achieve a 16-fold increase in Polymyxin B recovery compared with chitosan microparticles. This significant improvement is attributed to enhanced electrostatic interactions and the formation of distinct core/shell structures, as confirmed by fluorescence labeling. Specifically, the negative charge of the carboxylic groups in alginate interacts electrostatically with the positive charge of the ammonium groups in Polymyxin B. This interaction results in a more stable structure, higher encapsulation efficiency (EE), and a denser, more sustained release^53,54^

Sodium alginate, with its appropriate molecular weight, forms layered structures that facilitate a sustained drug release rate (26 ± 3% at 24 h), in contrast to the rapid release from chitosan formulations. We found that alginate concentrations ranging from 0.1% to 0.3% w/v increase particle sizes and encapsulation efficiency, indicating a positive correlation between alginate concentration and EE. Additionally, the molecular weight and viscosity of alginate solutions contribute to the formation of these layered structures, thereby enhancing the sustained release profile.^29,55^

Surfactants play a crucial role in the emulsification-gelation and extrusion processes by reducing interfacial tension and preventing droplet coalescence, which ensures the production of discrete microspheres. In contrast, chitosan spray drying methods involve dissolving chitosan in aqueous acetic acid and atomizing the solution in a hot air stream. Chitosan particles exhibit more variability and less stability due to pH and surface charge effects compared to alginate, affecting the overall recovery and release kinetics of Polymyxin B.^44,46,56^

### 3.10 Antimicrobial Formulation Testing Methodology for MIC, MBC, and Controls

In the goal of developing highly potent antimicrobial formulations, this study explored the impact of key excipients, specifically, alginate, chitosan, poloxamer 407, and HPβCD on the antimicrobial characteristics of ALG_H_CD_H_P_L_^PM^, ALG_L_CD_H_P_L_^PM^, CS_L_CD_H_P_L_^PM^, and CS_H_CD_H_P_L_^PM^. Minimum inhibitory concentration (MIC) and minimum bactericidal concentration (MBC) values were used to gauge the concentration needed to inhibit bacterial growth and achieve bactericidal effects against *E. coli* and *P. aeruginosa*.^59^ The selection of *E. coli* and *P. aeruginosa* for the study on polymyxin microparticles was guided by their clinical relevance as common pathogens involved in various infections, including nosocomial ones, and their known levels of antibiotic resistance, particularly to polymyxins. These two microbes are well-established models in microbiological and pharmacological research, providing a robust framework for experimental design and data interpretation.^43,46^ Additionally, their use leverages prior successful studies ensuring continuity and comparability in research.^4,60−62^

**Figure 6:**
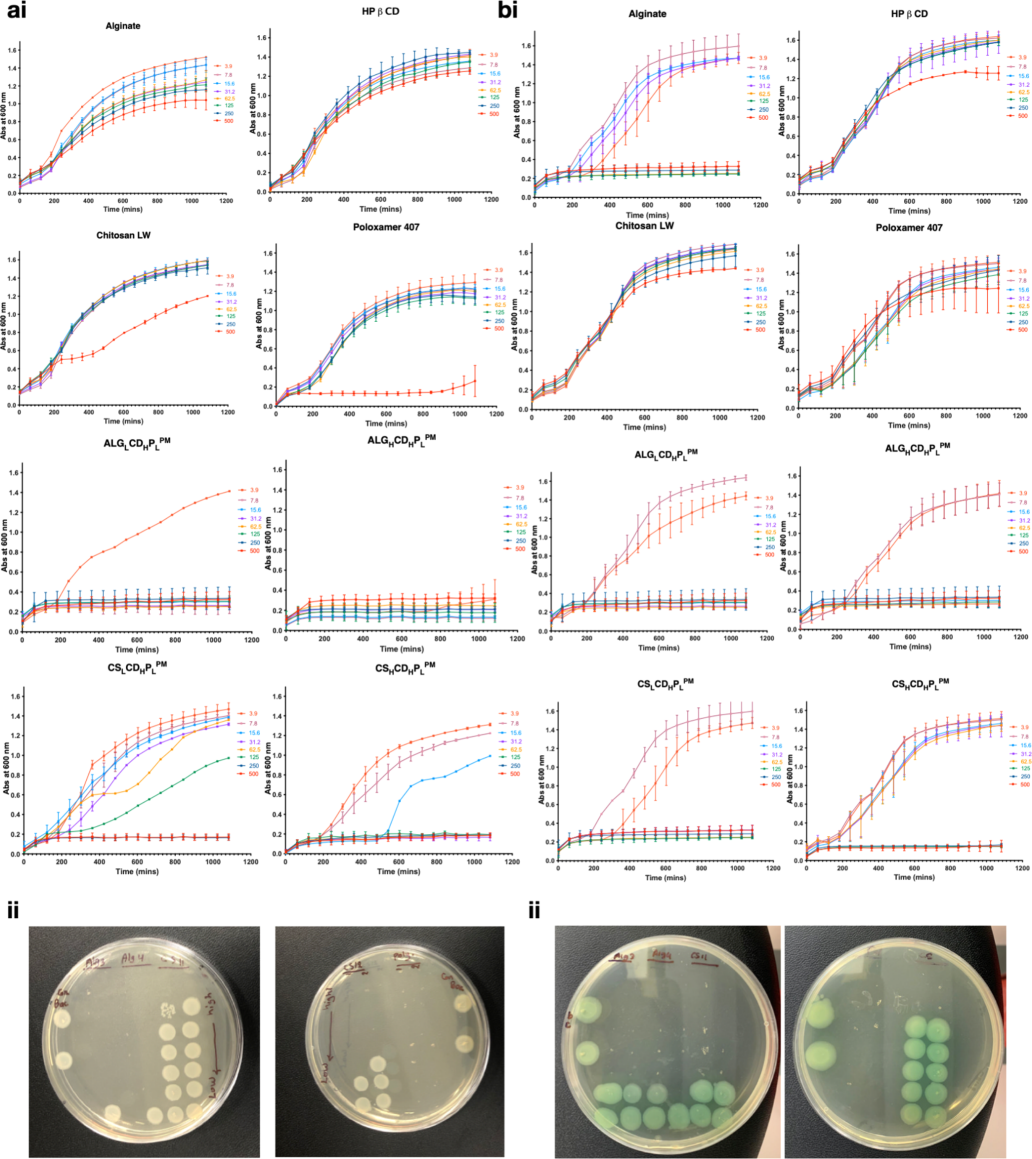
Microbial assessment. MIC and MBC assays conducted on the investigated formulations against *E. coli* (ai,ii) and *P. aeruginosa* (bi,ii). Polymyxin functions as a positive control with excipients acting as a negative control.

Comparisons were made using pure Polymyxin B as a positive control and the additives as a negative control (Figure 6a for *E. coli* and Figure 6b for *P. aeruginosa*). This experimental setup allowed for a thorough analysis and comparison of the effects of the additives in a microbiological context.^63^ As shown in Figure 6ai, *E. coli* exhibited a significant decline when exposed to ALG_H_CD_H_P_L_^PM^ at all tested concentrations, including the lowest tested at 3.8 μg/mL. ALG_L_CD_H_P_L_^PM^, CS_L_CD_H_P_L_^PM^, and CS_H_CD_H_P_L_^PM^ effectively suppressed bacterial growth at concentrations higher than 7.8, 250, and 31.2 μg/mL, respectively. Notably, CS_L_CD_H_P_L_^PM^ was less effective against *E. coli* compared to other studied formulations, potentially due to the low ratio of CS in the formulation. Conversely in Figure 6bi, *P. aeruginosa* exhibited some recovery in bacterial counts after 24 h with ALG_L_CD_H_P_L_^PM^, ALG_H_CD_H_P_L_^PM^, and CS_L_CD_H_P_L_^PM^ at concentrations lower than 15.6 μg/mL. This may be attributed to peptide degradation. On the other hand, CS_H_CD_H_P_L_^PM^ inhibited bacterial growth only at high concentrations from 125 up to 500 μg/mL, possibly due to the interaction between the negatively charged biofilm and the high positive charge of chitosan in CS_H_CD_H_P_L_^PM^.^63^ However, a significantly suppressed bacterial growth was observed up to 7 h of *P. aeruginosa* exposure to CS_H_CD_H_P_L_^PM^. Among the formulations studied, ALG_L_CD_H_P_L_^PM^ emerged as the most effective against both *E. coli* and *P. aeruginosa*. Notably, CS_H_CD_H_P_L_^PM^ exhibited limited efficacy against *P. aeruginosa*, contrasting sharply with the pronounced inhibitory effects observed for other studied formulations. Additionally, the study highlighted the minimal antimicrobial activity of each component alone, emphasizing that specific components within the formulations were partly responsible for the observed effects. Alginate, identified as a potent contributor to antimicrobial activity, demonstrated remarkable efficacy at a low concentration (62.5 μg/mL) against *P. aeruginosa* as shown in Figure 6bii.

This underscores the significance of alginate in enhancing the formulations’ antimicrobial properties in ALG_H_CD_H_P_L_^PM^, which contained the highest ratio of alginate compared to the other studied formulations. Recognized for its biocompatibility and mucoadhesive properties, alginate likely plays a pivotal role in improving drug stability and release kinetics. The inclusion of poloxamer 407, a surfactant, played a role in boosting the antibacterial effectiveness of the microparticles by improving drug solubility and dispersion. Figure 6aii illustrates the antimicrobial efficacy of poloxamer 407 at a concentration of 500 μg/mL against *E. coli*. Meanwhile, HPβCD, the “glass cage”, known for forming inclusion complexes with drug molecules, likely shielded the antibiotic peptide, enhancing its stability in the aqueous environment and, consequently, its efficiency. This underscores how excipients not only aid in drug delivery but also significantly impact therapeutic outcomes.^64^

The congruence between MIC and MBC results is noteworthy, as illustrated in Figure 6aii and bii. This agreement indicates that the studied microparticles not only inhibit bacterial growth but also effectively kill the bacteria.^18^

**Figure 7:**
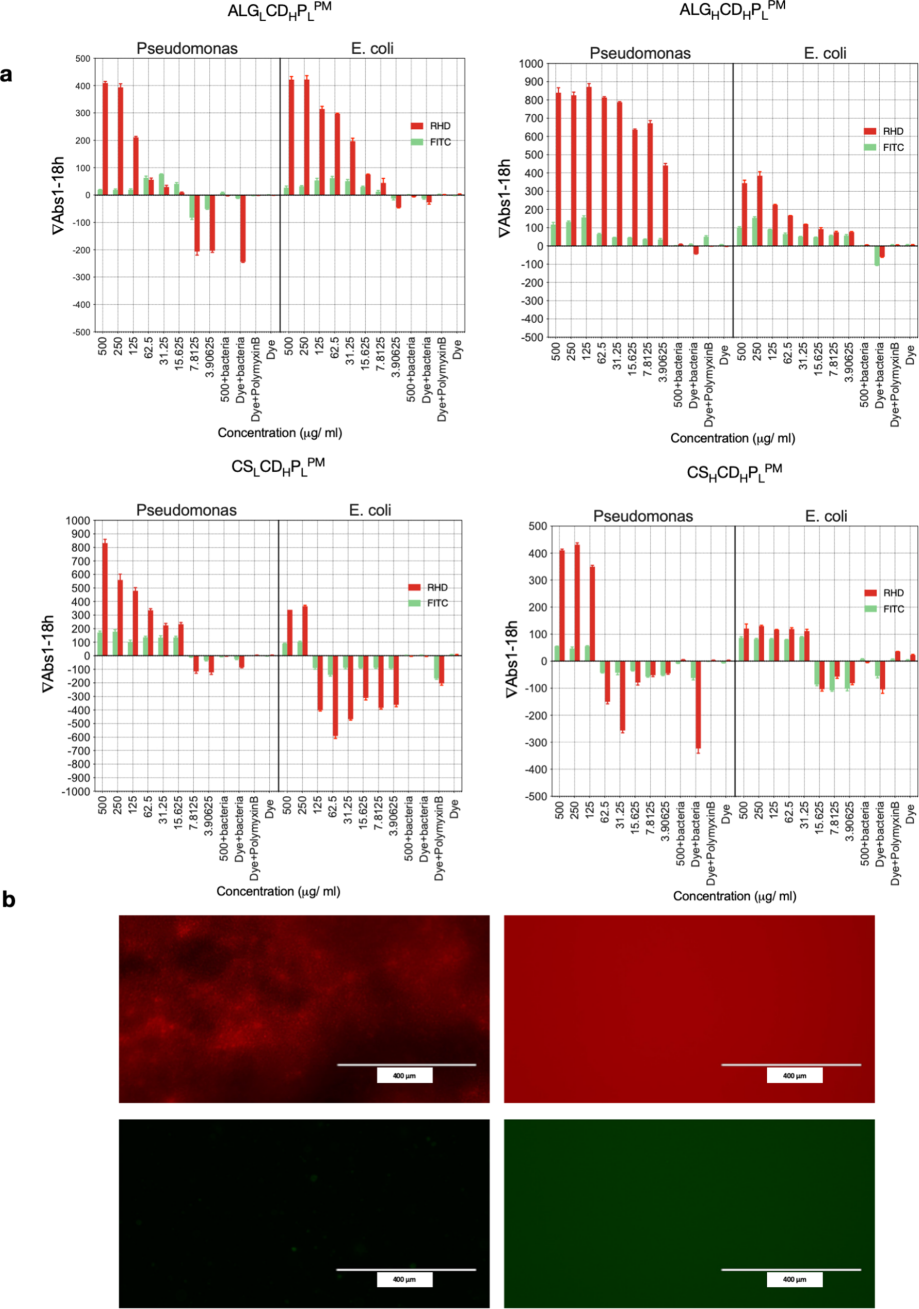
Dynamic fluorescence changes in the investigated formulations following microbial responses. (a) Vibrant fluorescence changes post treatment of *E. coli* and *P. aeruginosa* using the prepared microparticles, with FITC as the core label and Rhodamine B as the shell label. (b) Microscopic view unveils the fluorescence signal under a fluorescence microscope.

Achieving both growth inhibition and bacterial eradication is pivotal in antimicrobial therapy as it ensures complete suppression of the infection. These findings hold promise for the development of highly effective antimicrobial delivery systems, especially in the context of respiratory infections where targeted and efficient drug delivery is crucial.^18^Figure 7a illustrates the dynamic fluorescence response after treating *E. coli* and *P. aeruginosa* with the studied formulations, considering FITC as the core label and Rhodamine B as the shell label. Notably, rapid increases in fluorescence signals occur at bactericidal concentrations of ALG_L_CD_H_P_L_^PM^ (15.6 and 7.8), ALG_H_CD_H_P_L_^PM^ (3.9 and 3.9), CS_L_CD_H_P_L_^PM^ (15.62 and 250), and CS_H_CD_H_P_L_^PM^ (125 and 31.25) μg/mL for *P. aeruginosa* and *E. coli*, respectively.

Fluorescence values increased between 1 and 18 h, indicating a clear correlation between bacterial mortality and fluorescence release kinetics from the microparticles.^65^ In Figure 7b, a fluorescence signal is depicted under a fluorescence microscope. The rhodamine filter shows distinct visibility of microparticles, undergoing a striking transition: after 18 h, the complete release of rhodamine transforms the area into vibrant red fluorescence. Conversely, under the FITC filter, the region initially appears colorless, only revealing distinct fluorescence after the full release of FITC from the particles. This observation emphasizes the specificity and sensitivity of the FITC filter, capturing the precise moment of complete FITC release, the core material, after the interaction between bacteria and microparticles.^66^

Notably, pure Polymyxin B with dye shows no differences in the fluorescence signals before and after incubation. However, when bacteria were incubated with dye, a noticeable decrease in fluorescence signal occurred due to bacterial turbidity, impeding the fluorescence signal. No significant disparities were noted in fluorescence signals when a dye or microparticles without bacteria were used as controls. Employing innovative formulations and excipients can significantly boost the therapeutic effectiveness of Polymyxin, overcoming clinical limitations in treating Gram-negative bacterial infections. ALG_L_CD_H_P_L_^PM^, ALG_H_CD_H_P_L_^PM^, and CS_L_CD_H_P_L_^PM^ emerge as promising candidates for combating *P. aeruginosa* infections, backed by MIC and MBC values, with a warning against using CS_H_CD_H_P_L_^PM^ for this purpose.^66^

### 3.11 Biofilm Formation

Our study investigated whether formulations could prevent biofilm formation of *P. aeruginosa*, a major concern in respiratory tract infections.^67^ A crucial regulator of biofilm formation in *P. aeruginosa* is the second messenger signaling molecule bis(3′5′) cyclic dimeric guanosine monophosphate (c di GMP).^68^ Elevated cd GMP levels in *P. aeruginosa* are associated with biofilm formation, contributing to virulence and bacterial persistence in hosts. Compounds targeting c di GMP to reduce and prevent biofilm formation are gaining attention.^67,69^

**Figure 8:**
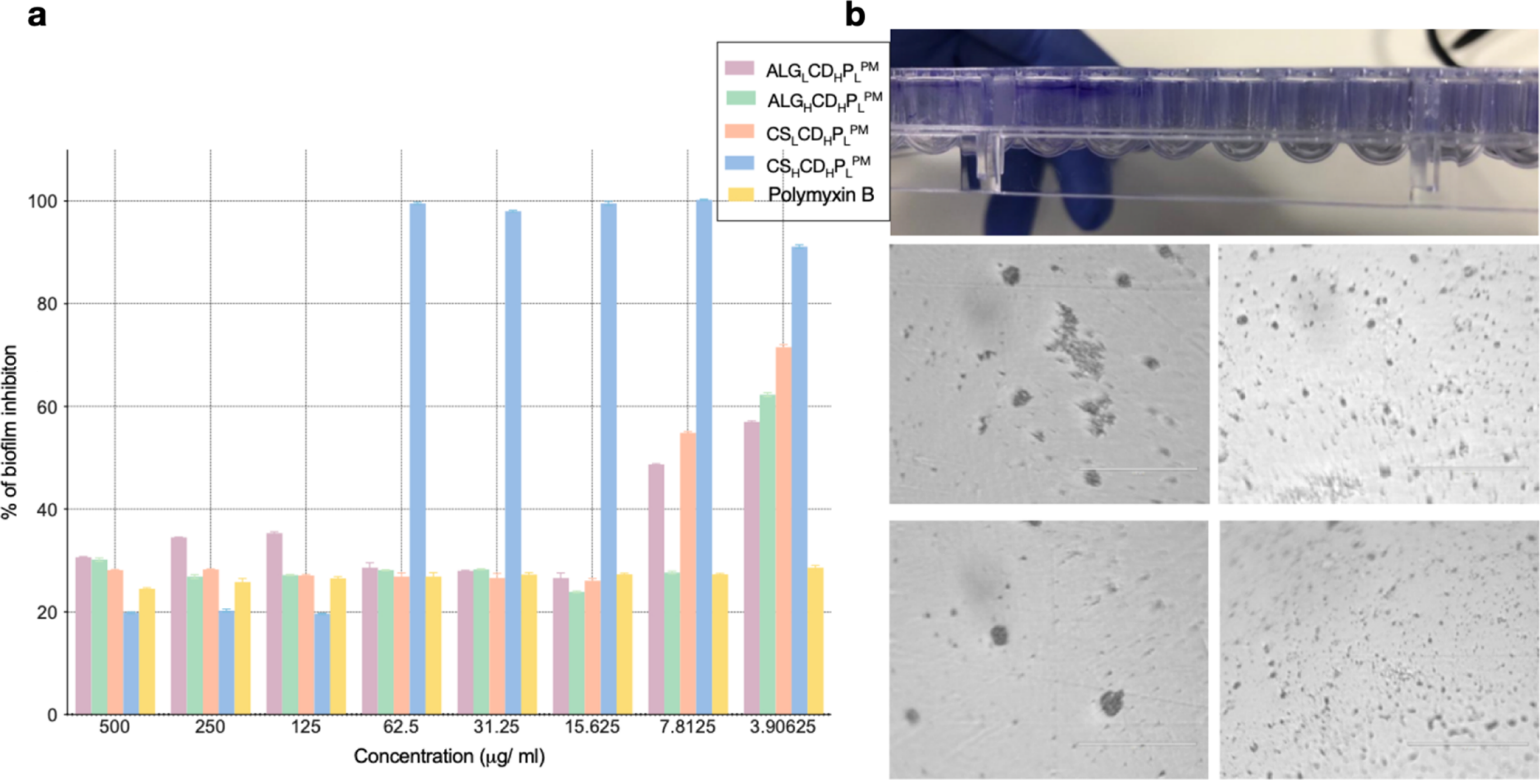
Dynamic fluorescence changes in investigated formulations following microbial responses. (a) Vibrant fluorescence changes post treatment of *E. coli* and *P. aeruginosa* using the prepared microparticles, with FITC as the core label and Rhodamine B as the shell label. (b) Microscopic view unveils the fluorescence signal under a fluorescence microscope.

The assessment revealed that ALG_H_CD_H_P_L_^PM^ and ALG_L_CD_H_P_L_^PM^ exhibited similar efficiency to pure Polymyxin B in reducing the survivability of biofilm formation in *P. aeruginosa* at all studied concentrations from 3.9 to 500 μg/mL. CS_L_CD_H_P_L_^PM^ showed a significant reduction in biofilm viability at concentrations higher than 7.8 μg/mL, while CS_H_CD_H_P_L_^PM^ was effective only with 125, 250, and 500 μg/mL as shown in Figure 8a. Figure 8b shows images of vials showing the crystal violet taken up by biofilms formed at the air–water interface after removing the aqueous phase, with the residual purple coloration indicating dye uptake by bacteria.^47,70^

Polymyxins exert their bactericidal effects through diverse mechanisms including membrane disruption, inhibition of bacterial respiration, generation of reactive oxygen species (ROS), ribosome binding, and modulation of cell division dynamics. These multifaceted mechanisms underscore the complexity of polymyxin activity and its effectiveness against bacterial pathogens.^29^ Maintaining the structural integrity of polymyxin molecules is crucial for ensuring their efficacy across these various pathways, as any degradation or modification could compromise their ability to interact with bacterial targets and exert their bactericidal effects effectively.^59,61,71^ Comparison with blank excipients lacking antimicrobial activity provides evidence of the stability of our formulation, further supporting its potential for therapeutic use.

The toxicity of polymyxin formulations is a significant concern due to their known nephrotoxic and neurotoxic effects. Encapsulating polymyxin in a polymeric carrier has the potential to reduce this toxicity.^4^ Polymeric carriers can provide a controlled release of the drug, which may decrease the peak plasma concentrations and, therefore, reduce the associated toxic effects. Additionally, encapsulation can target the delivery of polymyxin more specifically to the site of infection, thereby limiting systemic exposure and minimizing adverse effects. This strategy not only enhances the therapeutic index of polymyxin but also offers a promising approach to improving its safety profile in clinical use.^5,43,59,72^

## 4 Conclusions

In summary, the application of the 3FN spray drying process for microencapsulation emerges as a highly promising technique for Polymyxin B. Particularly, ALG_H_CD_H_P_L_^PM^, with its higher alginate ratio, demonstrates exceptional Polymyxin B recovery. Morphological assessments confirm the successful formation of microparticles with core/shell structures. FTIR spectra and XRD analyses reveal specific hydrogen bonds between Polymyxin B and other components in the formulations, maintaining essential functional groups in an amorphous state. The incorporation of release analyses provides further insights into the structural characteristics of the microparticles, validating the sustained release of Polymyxin B post spray drying. Moreover, preliminary assessments of the antimicrobial activity of the formulations demonstrate the remarkable efficacy of ALG_H_CD_H_P_L_^PM^ in preventing *P. aeruginosa* biofilm formation and inhibiting bacterial growth in both *E. coli* and *P. aeruginosa*, even at low concentrations of 3.8 μg/mL. In essence, this investigation offers valuable insights into advancing sophisticated drug delivery systems, particularly for critical antibiotics such as Polymyxin B. It establishes a comprehensive framework to address challenges related to stability, dosing precision, and antimicrobial efficacy, positioning it as a promising candidate for lung delivery.

## Supporting Information Available

Thermogravimetric analysis (TGA) results for all formulations; TGA data for both alginate-based and chitosan-based formulations, demonstrating weight loss profiles across the temperature range of 30 to 150 °C, and results highlight the presence of residual humidity and water of hydration within the microparticle formulations (PDF)
