# Supplementary material for: Innovative Microencapsulation of Polymyxin B for Enhanced Antimicrobial Efficacy via Coated Spray Drying
Source: Mol Pharm. 2024 Oct 8;22(1):113–30. doi: 10.1021/acs.molpharmaceut.4c00594 (PMC11707731; doi:10.1021/acs.molpharmaceut.4c00594)
Supplement: Supplementary file 1 — mp4c00594_si_001.pdf [file mp4c00594_si_001.pdf]

Innovative microencapsulation of Polymyxin B for enhanced antimicrobial efficacy via coated spray drying

Amal Yousfan[a], Arwa Omar Al Khatib[a,b], Afrah M H Salman[c,d], Mahmoud H. Abu Elella[a], Glyn Barrett[c], Nicholas Michael[e], Mohammed Gulrez Zariwala[f], Hisham Al Obaidi[a]*

[a]School of Pharmacy, University of Reading, Reading RG6 6AD, UK
[b]Faculty of Pharmacy, Al Ahliyya Amman University, Amman 19111, Jordan
[c]School of Biological Sciences, University of Reading, Reading RG6 6AD, UK
[d]College of Pharmacy, Pharmacology and Toxicology department, Mustansiriyha University, , Baghdad, Iraq
[e]Chemical Analysis Facility, University of Reading, Reading RG6 6AD, UK
[f]Centre for Nutraceuticals, School of Life Sciences, University of Westminster, 115 New, Cavendish Street, London W1W 6UW, UK

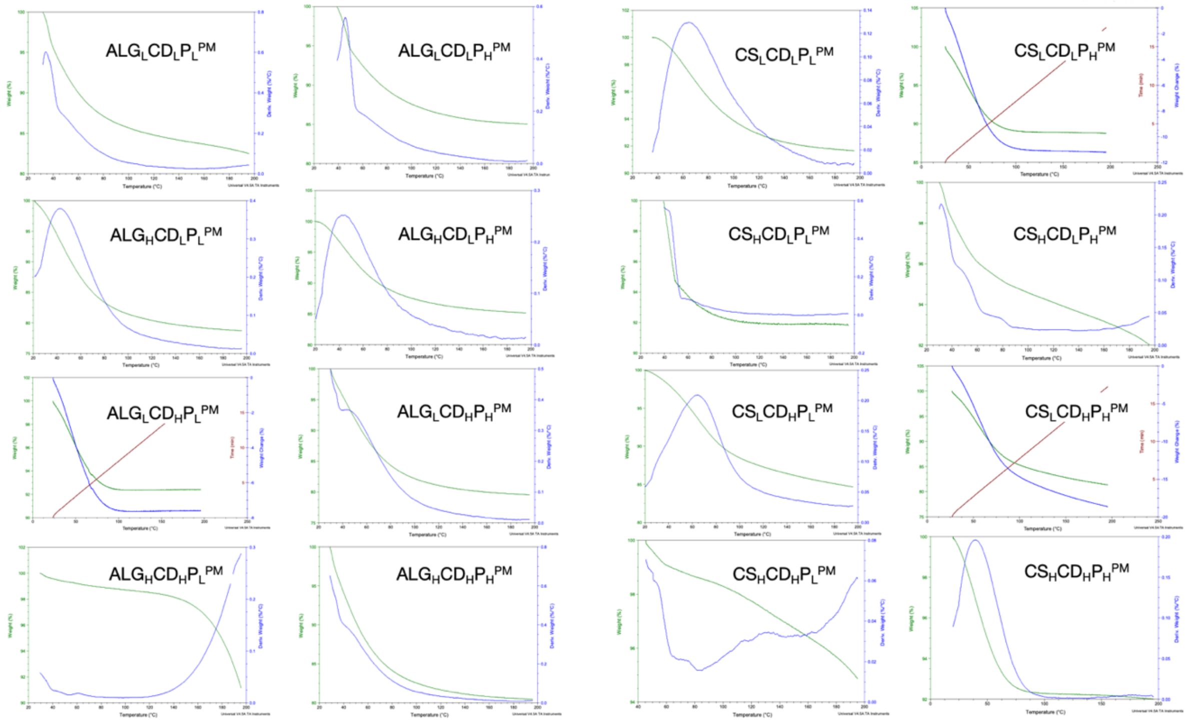

Figure S1. Thermogravimetric Analysis (TGA) results for all formulations. The supplementary information includes TGA data for alginate formulations and chitosan formulations, illustrating weight loss across the temperature range of 30°C to 150°C, indicating residual humidity and water of hydration.
